# Evaluation of the Diagnostic Accuracy of Serum D-Dimer Levels in Pregnant Women with Adnexal Torsion

**DOI:** 10.3390/diagnostics5010001

**Published:** 2015-01-05

**Authors:** Hasan Onur Topçu, Can Tekin İskender, Ufuk Ceran, Oktay Kaymak, Hakan Timur, Dilek Uygur, Nuri Danışman

**Affiliations:** Zekai Tahir Burak Women’s Health Education and Research Hospital, 1549 Cadde, Hardem Apartmanı, B Blok, Daire 12 Çiğdem-Çankaya, Ankara 06300, Turkey; E-Mails: c_iskender@yahoo.com (C.T.I.); mehmet.ufuk.ceran@gmail.com (U.C.); oktaykaymak@hotmail.com (O.K.); drhakantimur@gmail.com (H.T.); dilekuygur@gmail.com (D.U.); nuridanisman@gmail.com (N.D.)

**Keywords:** adnexal torsion, D-dimer, ovarian torsion, pregnancy

## Abstract

We aimed to evaluate the diagnostic accuracy of serum D-dimer levels in pregnant women with adnexal torsion (AT). The pregnant women with ovarian cysts who suffered from pelvic pain were divided into two groups; the first group consisted of the cases with surgically proven as AT (*n* = 17) and the second group consisted of the cases whose pain were resolved in the course of follow-up period without required surgery (*n* = 34). The clinical characteristics and serum D-dimer levels were compared between the groups. Patients with AT had a higher rate of elevated serum white blood cell (WBC) count (57%* vs.* 16%, *p* = 0.04) and serum D-dimer levels (77%* vs.* 21%, *p* < 0.01) on admission in the study group than in the control group. Elevated D-dimer and cyst diameter larger than 5 cm yielded highest sensitivity (82% for each); whereas the presence of nausea and vomiting and elevated CRP had the highest specificity (85% and 88%, respectively). This is the first study that evaluates the serum D-dimer levels in humans in the diagnosis of AT, and our findings supported the use of D-dimer for the early diagnosis of AT in pregnant women.

## 1. Introduction

Adnexal torsion (AT) is a gynecologic emergency as the salvage of the involved adnexa depends on prompt recognition and surgical intervention. AT during pregnancy is relatively rare with an incidence of 1–10 per 1000 pregnancies [1]. It is usually confined to the first half of pregnancy with approximately 80% of cases occurring within the first 20 weeks of pregnancy [2]. Neither physical nor sonographic and Doppler findings alone are accurate in the diagnosis of AT. To improve diagnostic accuracy, a scoring system was proposed in a non-pregnant population previously [3]. Previous data have shown that, each of the components of the scoring system such as nausea and vomiting, presence of an ovarian cyst, presence of peritoneal signs and leukocytosis are also valuable in pregnant populations [2,4]. However, diagnosis of AT in pregnant populations can be more challenging as these findings may be attributed to pregnancy related complications. In addition, the diagnostic value of each finding has not been adequately investigated for pregnant populations.

D-Dimers, specific cross-linked fibrin derivatives produced when fibrin is degraded by plasmin, are extremely sensitive indicators of both intravascular and extravascular thrombosis [5]. Pregnant women comprise an extremely heterogeneous population with respect to their baseline risk for DVT or PTE; so D-dimer measurements are currently not recommended in the pregnant population for the diagnosis of deep venous thrombosis (DVT) or pulmonary thromboembolism (PTE) as there are no adequate trials that show well-established reference ranges for gestational age [6]. However, recent efforts have aimed to establish gestational age specific reference ranges for serum D-dimer levels [5,7,8].

The partial or complete obstruction of the adnexa may cause ischemia and ovarian necrosis. We proposed that serum D-dimer may be increased in the intestinal ischemia and venous thromboembolic event of the ovary. Therefore, we aimed to investigate the diagnostic accuracy of the serum D-dimer levels in pregnant women with AT.

## 2. Materials and Methods

The present study retrospectively reviewed cases of AT during pregnancy between June 2009 and June 2014 at the high risk perinatology clinic of Zekai Tahir Burak Women Health Research and Training Hospital. The patients who were admitted with pelvic pain and ovarian cyst were hospitalized in the early pregnancy unit of our perinatology clinic. The diagnostic algorithm in these patients includes complete blood count, liver and kidney function blood tests, hemostasis profile, serum C-reactive protein analysis, urine and vaginal cultures. The physical examination included abdominopelvic examination to check for the presence of peritoneal irritation signs, vaginal bleeding or discharge, uterine tenderness and fever.

Ultrasonography with Doppler analysis was performed in all patients via transvaginal or transabdominal route depending on gestational age by Voluson 730 PRO 4D device (General Electric Medical Systems, Milwaukee, WI, USA).

The diagnosis was confirmed by laparotomy or laparoscopy in all patients with adnexal torsion. In our tertiary hospital, 75,000 live births had occurred in the study period. The study group consisted of 17 pregnant women with AT whose diagnosis were confirmed surgically (*n* = 17) and control group consisted of the next two patients who admitted with pelvic pain and ovarian cyst who were hospitalized with a presumptive diagnosis of AT (*n* = 34). The diagnosis of AT was excluded in these patients whose symptoms were subsided or whose diagnosis was changed after hospital admission.

The charts of the patients were reviewed and the following data were obtained; age, gestational age at admission, parity, presence of controlled ovarian stimulation, history of AT, diameter of adnexal cyst and the presence of negative flow in Doppler, serum white blood cell count (WBC), serum C-reactive protein (CRP) and D-dimer levels at admission. Clinical signs and symptoms including the presence of acute onset of pain (less than 8 h), peritoneal signs, nausea and vomiting were obtained from medical records.

Serum D-dimer levels were obtained on the day of admission from the antecubital vein and collected into tubes containing 0.5 mL of 38 g/L sodium citrate. For INNOVANCE D-dimer (Siemens Healthcare Diagnostics, GmbH, Marburg, Germany) was used as reagents similarly to a previously published article from our hospital [9]. The gestational specific reference ranges were used to evaluate elevated levels [7]. The surgical route, type of procedure carried out, final histopathological diagnosis and the pregnancy outcome of patients retrieved from medical records.

Approval was obtained from ethics and the education issues coordinating committee of Zekai Tahir Burak Women Health Research and Training Hospital. Statistical analysis was performed using SPSS version 18 (Statistical Package for the Social Sciences, Chicago, IL, USA). Student’s *t*-test was performed for parametric variables between groups that distribute normally, for parametric variables without normal distribution; Mann-Whitney *U* test was performed. Fisher’s exact test and Chi-square test was performed for non-parametric variables between groups where appropriate. Sensitivity, specificity, positive and negative predictive values and positive and negative likelihood ratios were calculated for diagnostic signs and symptoms. A P value less than 0.05 was considered as significant.

## 3. Results

In the study period, there were 75,000 live births in our tertiary hospital and we detected 17 AT in pregnant women in this period. The incidence of the AT in pregnant women was found 4.4/10,000. Clinical characteristics and pregnancy outcome of patients are shown in Table 1. The rate of nulliparity (88% *vs.* 41%, *p* < 0.01) and controlled ovarian hyperstimulation (41% *vs.* 15%, *p* < 0.01) were higher in the study group than in the control group. The diameter of the cyst was significantly larger in the study group than in the control group (7.5 ± 3.2 cm *vs.* 4.9 ± 1.6 cm, *p* < 0.01; respectively). Negative flow in Doppler was found statistically higher in study group (8 (47.1%) *vs.* 2 (5.8%), *p* = 0.001). Other clinical characteristics were similar among the groups. The operative procedure and final histopathological diagnosis in patients with AT are depicted in Table 2 and Table 3. In 47% cases, histopathological diagnoses of the specimens were corpus luteum cysts or follicular cysts. Laparoscopy was the initial choice of operation in 15 patients (88%) while laparotomy was preferred in one patient. Conversion to laparoscopy was necessary in a single patient (6%). In 11 patients, the surgical procedure was adnexal detorsion alone (*n* = 6, 35.3%) or along with cystectomy (*n* = 5, 29.4%). In two patients (11.8%) ipsilateral salpingo-oophorectomy was performed and in one patient (5.9%) cystectomy and ovarian wedge resection was performed.

**Table 1:** Comparison of clinical characteristics and pregnancy outcome between groups.

Variables	Adnexal Torsion (*n* = 17)	Control (*n* = 34)	*p*
Age (years)	25.4 ± 4.6	26.0 ± 5.6	0.67
Nulliparity	15 (88%)	14 (41%)	**<0.01**
Controlled ovarian hyperstimulation	7 (41%)	5 (15%)	0.04
Multiple pregnancy	3 (18%)	1 (3%)	0.10
*Gestational period at admission*			
1st trimester	11 (65%)	18 (53%)	-
2nd trimester	6 (35%)	16 (47%)	0.42
3rd trimester	-	-	-
Cyst Diameter (cm)	7.5 ± 3.2	4.9 ± 1.6	**<0.01**
WBC (×10^3^) (cells/mm3)	13.3 ± 6.9	10.3 ± 2.8	0.19
*D-dimer (µg/mL)*			
Independent from trimester	1.83 ± 1.1	0.66 ± 0.37	**<0.01**
1st trimester	2.13 ± 1.1	0.60 ± 0.43	**<0.01**
2nd trimester	1.27 ± 0.8	0.73 ± 0.29	0.20
Pregnancy outcome *			
Term delivery	10 (63%)	28 (85%)	0.14
Preterm delivery	3 (19%)	3 (9%)	0.38
Abortion	3 (19%)	2 (6%)	0.31

Values are given as mean ± standard deviation or number (percentage), *p* < 0.05 = statistically significant. * excluding cases with elective termination of pregnancy.

**Table 2:** Details of the operative procedure in patients with adnexal torsion during pregnancy.

Operative Procedure	Number of Patients	Percentage (%)
Adnexal detorsion alone	6	35.3
Adnexal detorsion with cyst aspiration and IPL fixation	3	11.8
Adnexal detorsion with cystectomy	5	5.9
Adnexal detorsion with ipsilateral salpingectomy	2	5.9
Adnexal detorsion with cystectomy and ovarian wedge resection	1	5.9

IPL: Infundibulopelvic ligament.

**Table 3:** Final histopathological diagnosis in patients with adnexial torsion during pregnancy.

Pathological Diagnosis	Number of Patients	Percentage (%)
Corpus luteum cyst	6	35.3
Follicular cyst	2	11.8
Mature Cystic teratoma	1	5.9
Mucinous cystadenoma	1	5.9
Tubal ectopic pregnancy	1	5.9
No specimen obtained	6	35.3

Patients with AT had a higher rate of elevated serum WBC count (47.1% *vs.* 17.6%, *p* = 0.04), serum D-dimer levels (76.5% *vs.* 8.8%, *p* < 0.01) and nausea and vomiting (35.3% *vs.* 8.8%, *p* = 0.04) on admission in the study group than in the control group. However, the rates of elevated CRP, acute onset pain, and presence of peritoneal signs were similar (Table 4).

The diagnostic performance of physical findings is shown in Table 5. Elevated D-dimer and cyst diameter larger than 5 cm yielded highest sensitivity (82% for each); whereas the presence of nausea and vomiting and elevated CRP had the highest specificity (85% and 88%, respectively). The specificity, positive and negative predictive values of elevated serum D-dimer were 79%, 66% and 90%, respectively. Positive and negative likelihood ratios for D-dimer were 4.0 (95% confidence interval: 2.0–8.02) and 0.22 (0.08–0.63).

**Table 4:** Comparison of clinical and laboratory features among groups.

Variables	Adnexal Torsion (*n* = 17)	Control (*n* = 34)	*p*
Acute onset pain (<8 h)	11 (64.7%)	16 (47.1%)	0.23
Cyst diameter > 5cm	14 (82.4%)	14 (41.2%)	<0.001
Peritoneal signs	9 (52.9%)	13 (38.2%)	0.32
Nausea and vomiting	6 (35.3%)	3 (8.8%)	0.04
Negative flow in Doppler	8 (47.1%)	2 (5.8%)	0.001
Leukocytosis (WBC ≥ 12 × 10^3^) (cells/mm^3^) 10 (71.4%)	8 (47.1%)	6 (17.6%)	0.04
Elevated CRP (≥8 mg/L)	5 (29.4%)	4 (11.7%)	0.14
* Elevated D-dimer	13 (76.5%)	3 (8.8%)	0.001

Values are given as number (percentage), *p* < 0.05 = statistically significant. WBC: white blood cell, CRP: C-reactive protein, h: hours, * reference [7] was used to determine elevated levels.

**Table 5:** Comparison of clinical features in pregnant patients with adnexal torsion.

Variables	Sensitivity	Specificity	PPV	NPV	LR +	LR −
	% (95% CI)	% (95% CI)	% (95% CI)	% (95% CI)	(95% CI)	(95% CI)
Acute onset pain (<8 h)	65 (38–86)	53 (35–70)	41 (22–61)	75 (53–90)	1.4 (0.83–2.3)	0.67 (0.33–1.37)
Cyst diameter > 5cm	82 (56–96)	59 (41–75)	50 (31–69)	87 (66–97)	2.0 (1,26–3.16)	0.30 (0.10–0.87)
Negative Doppler flow	35 (14–62)	76 (59–89)	43 (18–71)	70 (53–84)	1.5 (0.62–3.63)	0.85 (0.57–1.26)
Peritoneal signs	53 (28–77)	62 (44–78)	41 (21–63)	72 (53–87)	1.4 (0.75–2.6)	0.76 (0.43–1.35)
Nausea and vomiting	35 (14–62)	85 (69–95)	55 (24–83)	73 (56–85)	2.4 (0.85–6.75)	0.76 (0.52–1.11)
Leukocytosis (WBC ≥ 12 × 10^3^) (cells/mm^3^) 10 (71.4%)	47 (23–72)	82 (65–93)	57 (29–82)	76 (58–88)	2.7 (1.10–6.46)	0.64 (0.40–1.03)
Elevated CRP (≥8 mg/L)	24 (7–50)	88 (73–97)	50 (16–84)	70 (54–83)	2.0 (0.57–7.03)	0.87 (0.65–1.16)
* Elevated D-dimer	82 (50–93)	79 (76–98)	66 (43–85)	90 (73–98)	4.0 (2.0–8.02)	0.22 (0.08–0.63)

CI: confidence interval, PPV: Positive predictive value, NPV: Negative predictive value, LR+: Positive likelihood ratio, LR−: Negative likelihood ratio, h: hours, CRP: C-reactive protein, * reference [7] was used to determine elevated levels.

## 4. Discussion

Herein, we reported a study among pregnant women with ovarian cysts who were admitted to our tertiary referral hospital suffering from pelvic pain. We compared the clinical and laboratory features in pregnant patients with ovarian cysts whose diagnosis were surgically proven as adnexal torsion and whose diagnosis were confirmed as permanent pelvic pain responded to the medical therapy in the course of follow-up period. We determined that an increase of the serum D-dimer level was a novel promising finding in the pregnant patients for the diagnosis of AT.

The management of the pelvic pain in women with ovarian cysts is still a complicated situation due to the probability of the presence of AT because the clinicians should decide promptly to perform surgery or not, in order to preserve the ovarian reserve. In addition, this situation is more complicated in pregnant women with ovarian cysts due to its low incidence, and a likely false positive diagnosis and the likely adverse effects of the surgery to the fetus. AT in pregnancy is a rare clinical entity with an incidence of 1–5/10,000 in spontaneous pregnancy [1]. During the study period, we determined that approximately 75,000 live births had occurred at our hospital; and we detected AT in 17 of 75,000 pregnancies, with an incidence of 4.4/10,000 similar with Smorgick *et al.* [10]. The most common ovarian cysts in pregnancy were reported in order as dermoid cysts, serous cystadenoma, mucinous cystadenoma and low malign potential tumors (37.6%, 13%, 14.5%, 7.2%, respectively) [11]. In another study, the majority of the ovarian cysts removed at the second trimester were dermoids (37%), cystadenomas (24%), persistent corpus luteum cysts (20%), paraovarian cysts (5%) and endometriomas (5%) [12]. In our study, the final pathological diagnoses of the cysts which resulted in twisted ovary were corpus luteum cyst (54.5%), follicular cyst (18.2%), mature cystic teratoma (9.1%), mucinous cystadenoma (9.1%) and tubal ectopic pregnancy (9.1%).

Controlled ovarian stimulation (COS) protocols may raise the AT rate by creating multiple cystic lesions of the ovary. The incidence of the AT in the COS cycles rises from 6% to 16% [13,14]. Moreover, AT was found to be with a high ratio (49%) in pregnancies conceived through the COS cycles [10]. Seven of 17 (41%) patients in AT group and 5 of 34 (15%) patients in control group were conceived through COS protocols and this difference reached a significant value in our study, in compliance with previous studies [10,15]. Commonly, the AT had occurred in the first trimester of the pregnancy [10,16,17,18]. In this current report, AT occurred in 11 of 17 cases (65%) in the first trimester, and in 6 of 17 cases (35%) in the second trimester. There were no AT cases in the third trimester similar with a past report [19].

Goh *et al.* [11] reported 8.3% AT rate in 126 pregnant patients in whom ovarian cyst diameter equal or greater than 5 cm. In the previous studies, the diameter of the ovarian cysts greater than 5 cm was reported as one of the most important parameters for the occurrence of AT [20,21]. In current study, we had 14 of 17 (82.4%) pregnant patients in the study group and 14 of 34 (41.2%) pregnant patients in control group whose ovarian cyst diameter was greater than 5 cm. The differences of the diameter of the ovarian cysts between the groups were statistically significant. We also evaluated the pregnancy outcomes including term delivery, preterm delivery and miscarriage rates. We did not detect any increased risk of surgery to the pregnancy outcomes which is similar to a recent study [1].

In our study, nausea and vomiting and leukocytosis in AT group were found higher than in the control group. Elevated CRP, peritoneal irritation signs and acute onset pain were the laboratory and clinical parameters which were found to be similar in both groups. To achieve the differential diagnosis in pregnant women who had ovarian cysts and pelvic or abdominal pain; the peritoneal irritation signs, acute onset pain or elevated CRP were found to be useless in those women. Nausea and vomiting and leukocytosis were found to be beneficial clinical and laboratory parameters but they are nonspecific and inadequate to achieve the diagnosis. Negative flow in Doppler was determined in 8 of 17 (47.1%) in study group. Despite the utility of Doppler examination, the clinicians need more parameters to diagnose AT. Additionally in pregnant women, because although low complication rates were reported in the second trimester surgery, we believe that to make decision for surgery in pregnant women is harder than in non-pregnant women.

The twisting of the ovary and its surrounding tissue may result in congestion due to the prior obstruction of the venous return at first, and with the persistence of the obstruction, the partial or complete obstruction may cause ischemia and ovarian necrosis. D-dimer is a product of fibrin turnover and it rises quite quickly after a thromboembolic event and it has begun to be used widely after its discovery as an indicator of intestinal ischemia and venous thromboembolic disorders in all body organs, including the lungs, pelvis, upper extremities, thigh and calf [5]. To our knowledge, there is no report about the serum maternal D-dimer levels in pregnant women with AT. To preserve ovarian reserve in cases with AT, it is important to diagnose and to intervene early; thus a laboratory parameter that can support early diagnosis, such as; D-dimer, which may be more important for early diagnosis. In an experimental animal study, Kart *et al.* [22] demonstrated the increase of the D-dimer in AT. In our study, elevated D-dimer levels were detected significantly higher in AT group than in the control group (13 of 17, 76.5%, 3 of 34, 8.8%, respectively). We think that further studies with larger populations are needed to understand the diagnostic role of the serum D-dimer levels in pregnant women with AT.

Our study has several limitations. This is a retrospective study and the retrospective design of the study may affect some results; however, our perinatology clinic has strict rules for diagnostic algorithms, and these reduce the differences in the management of the cases. Our data depends on assay information; this is a limitation of our study. Our trial has a small number of the study population and a follow up D-dimer test may be performed to assess whether it will increase or decrease further; however, it is very difficult to design a prospective study for a disease which has a low incidence. There is also a limitation regarding the trials which investigate D-dimer levels due to the lack of the standardization on the assay. The D-dimer value may change by using a different assay methodology. Standardization may be required for the trials which evaluate the D-dimer levels. Some cases in the control group might have AT and it may be resolved spontaneously, so this is another limitation of our study, however, there is no certain way to diagnose spontaneously resolved ATs.

## 5. Conclusions

This is the first study that evaluates the serum D-dimer levels in humans in the diagnosis of AT, and our findings supported that the D-dimer may be used for the early diagnosis of AT in pregnant women.
